# Clinical Efficacy of Dienogest for Symptomatic Adenomyosis

**DOI:** 10.3390/jcm15072763

**Published:** 2026-04-06

**Authors:** Yejin Kwon, Ju Hee Kim, Hee Dong Chae, Sa Ra Lee, Sung Hoon Kim

**Affiliations:** Department of Obstetrics and Gynecology, University of Ulsan College of Medicine, Asan Medical Center, 88 Olympic-Ro 43-GIL, Songpa-gu, Seoul 05505, Republic of Koreaxjuheex@gmail.com (J.H.K.); hdchae@amc.seoul.kr (H.D.C.)

**Keywords:** adenomyosis, CA-125, dienogest, endometriosis, medical treatment

## Abstract

**Background:** This study was conducted to assess whether medical treatment with dienogest (DNG) is effective in women with symptomatic adenomyosis. **Methods:** This single-center, retrospective study included patients with symptomatic adenomyosis treated with oral DNG 2 mg daily. We evaluated the clinical symptoms, uterine volume, and serum CA-125 levels in these patients along with adverse events at baseline and after 3, 6, 12, 18, and 24 months of treatment, respectively. **Results:** A total of 102 patients were analyzed. Among women with dysmenorrhea, 79.5% reported improvement in dysmenorrhea, and the mean time to improvement in dysmenorrhea was 7.18 months. Improvement in heavy menstrual bleeding was observed in 88.3% of patients, with a mean time to improvement in heavy menstrual bleeding of 5.44 months. We could see that the uterine volume decreased significantly after 18 months of treatment. Serum CA-125 levels declined significantly from 3 months onward and remained reduced through 24 months. More than half of the patients (55.8%) continued DNG without complications, whereas 31.3% discontinued treatment or switched to alternative therapies. **Conclusions:** DNG effectively improved clinical symptoms and reduced uterine volume and serum CA-125 levels without serious adverse events in patients with symptomatic adenomyosis.

## 1. Introduction

Adenomyosis is a common benign gynecologic disorder characterized by the presence of ectopic endometrial glands and stroma within the myometrium, leading to reactive myometrial hypertrophy and hyperplasia. Although its true prevalence is difficult to determine due to diagnostic variability, adenomyosis is estimated to affect up to 20–35% of women of reproductive age, with higher prevalence reported among women in their late reproductive years [1]. The disease is increasingly recognized in younger women owing to improved imaging techniques and heightened clinical awareness. Clinically, adenomyosis is associated with dysmenorrhea, heavy menstrual bleeding (HMB), abnormal uterine bleeding (AUB), and chronic pelvic pain, all of which can significantly impair daily functioning and quality of life [1].

In addition to symptomatic burden, adenomyosis has been linked to infertility, subfertility, and adverse obstetric outcomes, including preterm birth, preeclampsia, and increased risk of cesarean delivery [2]. The pathophysiology of adenomyosis remains incompletely understood; however, proposed mechanisms include invagination of the endometrial basalis into the myometrium, metaplasia of Müllerian remnants, and tissue injury and repair processes [3]. These mechanisms are thought to be driven by estrogen dependence, chronic inflammation, and abnormal uterine peristalsis, resulting in progressive myometrial remodeling and symptom exacerbation [3].

Historically, adenomyosis was definitively diagnosed only after hysterectomy, as confirmation required histopathological examination of excised uterine tissue, thereby restricting diagnosis only to women who had completed childbearing or had severe symptoms warranting surgery. Over the past two decades, advances in imaging modalities have revolutionized the diagnostic approach. Transvaginal ultrasonography is widely used as a first-line diagnostic tool and typically reveals features such as uterine enlargement, asymmetric myometrial thickening, heterogeneous myometrial echotexture, myometrial cysts, and ill-defined endometrial–myometrial junctions [1]. Magnetic resonance imaging (MRI) offers higher diagnostic accuracy and reproducibility, with characteristic findings including junctional zone thickening greater than 12 mm on T2-weighted images, low-signal intensity areas with punctate high-signal foci within the myometrium, and a globular uterine configuration [4]. These advances have enabled earlier diagnosis and expanded the population of women eligible for conservative management. According to a systematic review, the pooled sensitivity and specificity for the diagnosis of adenomyosis were 72% and 81% for transvaginal ultrasound, and 77% and 89% for MRI, respectively, when histopathology was used as the reference standard [5].

Management strategies for adenomyosis are individualized and depend on symptom severity, disease extent, patient age, and reproductive plans. Although hysterectomy remains the definitive treatment, many patients prefer uterus-preserving options, particularly those desiring future fertility or seeking to avoid major surgery. Consequently, medical therapy constitutes the cornerstone of initial management. Available medical options include non-hormonal agents such as nonsteroidal anti-inflammatory drugs and tranexamic acid, as well as hormonal therapies including combined oral contraceptives (COCs), gonadotropin-releasing hormone agonists, and progestins [6]. Progestin-based therapies, including oral progestins, depot-medroxyprogesterone acetate, and the levonorgestrel-releasing intrauterine system (LNG-IUS), aim to counteract estrogen-driven proliferation and suppress inflammatory activity within adenomyotic lesions [6]. Although the evidence is experimental, nonsteroidal anti-inflammatory drugs are also a possible therapeutic option for endometriosis [7,8].

Dienogest (DNG) is an orally active synthetic 19-nortestosterone derivative with high selectivity for the progesterone receptor and minimal androgenic activity. It has been extensively studied and widely prescribed for the treatment of endometriosis [9]. DNG exerts antiproliferative, anti-inflammatory, and anti-angiogenic effects on ectopic endometrial tissue, leading to suppression of lesion growth, reduction in inflammatory mediators, and alleviation of pain-related symptoms [10,11,12,13,14,15]. Given the shared pathophysiological features between endometriosis and adenomyosis—particularly estrogen dependence, chronic inflammation, and aberrant tissue remodeling—DNG has been proposed as a promising therapeutic option for adenomyosis. Also, as the prevalence of adenomyosis was observed to be higher in patients with endometriosis, confirmation of therapeutic role of DNG in concomitant adenomyosis is important [16].

Several clinical studies have assessed the efficacy of DNG in patients with adenomyosis and have reported significant improvements in dysmenorrhea, chronic pelvic pain, and heavy menstrual bleeding. However, the available evidence remains inconsistent, largely due to heterogeneity in study design, small sample sizes, variations in outcome definitions, and relatively short follow-up periods. A recent meta-analysis demonstrated that DNG effectively controlled adenomyosis-related symptoms, particularly dysmenorrhea, chronic pelvic pain, and HMB, although no significant reduction in uterine volume was observed [13]. In contrast, another meta-analysis comparing DNG with LNG-IUS suggested that DNG is more effective in reducing pelvic pain and uterine volume [15]. Additional comparative studies have shown that DNG provides superior relief of dysmenorrhea compared with other medical treatments, including COCs and LNG-IUS [11]. Moreover, a large-scale meta-analysis confirmed that long-term DNG treatment was particularly effective in patients with severe dysmenorrhea, defined as a baseline visual analogue scale (VAS) score ≥7 [17]. Recent long-term follow-up in perimenopausal women with adenomyosis treated with DNG showed that a substantial proportion experienced effective pelvic pain and bleeding control, supporting its long-term clinical application [18].

Despite these encouraging findings, several clinically relevant questions remain unanswered. Patient responses to DNG vary widely, and concerns persist regarding adverse effects, treatment adherence, and long-term safety. In addition to that, a recent study suggested the functional progesterone resistance and resistance to progestin treatment in adenomyosis [19,20]. Furthermore, real-world data evaluating both symptom control and objective disease parameters, such as uterine volume and imaging findings, over extended treatment durations are limited. Therefore, this study aimed to evaluate the clinical efficacy and adverse outcomes of DNG in patients with symptomatic adenomyosis, with longitudinal assessment of symptoms, imaging parameters, and biochemical markers, to provide further evidence to inform individualized treatment strategies.

## 2. Materials and Methods

### 2.1. Study Design and Population

This single-center retrospective chart review included patients who were treated with DNG between 1 January 2020 and 30 November 2024 at Asan Medical Center. Eligible patients were women aged 20–55 years who were diagnosed with adenomyosis by ultrasonography or MRI and had a body mass index (BMI) ≥18 and <40 kg/m^2^. All participants presented with at least one adenomyosis-related symptom, including heavy menstrual bleeding, abnormal uterine bleeding, or chronic pelvic pain. Patients were required either to have no prior history of hormonal therapy or to have discontinued hormonal treatment at least one month before enrollment.

Patients were excluded if they had used hormonal contraceptives within one month prior to the screening date. The one-month interval was chosen to allow for adequate washout of prior hormonal contraceptives, thereby minimizing residual hormonal effects and enabling assessment of the independent effects of DNG. We also excluded patients currently using an LNG-IUS or a subdermal contraceptive implant, as well as those who had received GnRH agonists within the preceding three months. All participants in this study were premenopausal.

Additional exclusion criteria included a history of hypersensitivity, adverse reactions, or intolerance to DNG, the presence of uterine anomalies, uterine fibroids, prior uterine artery embolization or high-intensity focused ultrasound treatment, and the presence of malignancy.

### 2.2. Intervention

All patients received DNG 2 mg (Losanne^®^, Shin Poong Pharm, Republic of Korea) orally once daily.

### 2.3. Outcomes and Data Collection

Outcomes were assessed at baseline and after 3, 6, 12, 18, and 24 months of treatment, respectively. Evaluated clinical symptoms included dysmenorrhea, chronic pelvic pain, AUB, and HMB. Ultrasonographic measurements of anterior and posterior uterine wall thickness and uterine volume were obtained, calculated as length × width × height × 0.523 cm^3^ as previously described [21]. Serum CA-125 levels have been reported to be elevated in patients with adenomyosis compared with women with a normal uterus and may reflect inflammatory activity or disease burden. Therefore, CA-125 levels were assessed in the present study. Data on the serum CA-125 levels were also collected.

### 2.4. Statistical Analysis

To describe the distribution of variables, continuous variables were presented as mean ± standard deviation (SD) and Categorical variables were expressed as frequencies and percentages. We utilized general linear model (covariance pattern model in linear mixed model) to confirm the time effect on uterine volume, anterior and posterior uterine wall thickness, and CA 125 level. We used a general linear model to evaluate whether there was a significant change over time. Because measurements were repeatedly collected from the same patients, we applied a linear mixed model with a covariance pattern structure to appropriately account for the correlation among repeated observations within each individual.

The Cox proportional hazards model was used to identify factors associated with symptom improvement. The variables analyzed included age, BMI, parity, baseline symptom, accompanying endometriosis, CA 125, uterine length, uterine width, uterine anterior–posterior diameter, anterior uterine wall thickness, posterior uterine wall thickness, and uterine volume.

The Wilcoxon rank-sum test was used to analyze differences in symptom improvement between patients with adenomyosis with concomitant endometriosis and those without endometriosis. Spearman correlation analysis was used to evaluate whether symptom changes were associated with changes in imaging findings.

All statistical analyses were performed with the SAS version 9.4 (SAS Institute Inc., Cary, NC, USA). The *p*-value < 0.05 was considered to be statistically significant.

## 3. Results

### 3.1. Baseline Characteristics of the Participants

A total of 102 patients were included, with a mean age of 39.84 ± 7.01 years and a mean body mass index (BMI) of 22.63 ± 4.26 kg/m^2^. Over 50% of patients had no history of delivery. The most common symptoms were dysmenorrhea (76.47%) and HMB (58.82%). Concomitant or prior endometriosis was present in 43.14% (Table 1).

### 3.2. Previous Hormonal Treatment

Most patients (68.6%) used DNG as the first-line hormonal therapy. Previous hormonal treatments are presented in Table 2.

### 3.3. DNG Treatment Continuation

During the study period, 55.88% continued DNG without complications, whereas 31.37% changed to alternative therapy, including hysterectomy. The other 12.75% were lost to follow-up. When treatment was changed, hysterectomy was the most frequent alternative, followed by combined oral contraceptives (11.76%, 12 cases), levonorgestrel-releasing intrauterine system and GnRH agonists (3.92%, 2 cases each), and etonogestrel-releasing subdermal implant, sclerotherapy for concomitant endometrioma, and non-hormonal treatments (analgesics, tranexamic acid) (1.96%, 1 case each). Among primary reasons for treatment change included persistent HMB, dysmenorrhea or pelvic pain symptoms (28.12%) and aggravated or newly developed AUB (25.00%). Adverse events reported in single cases included hair loss, headache, depressive mood, pulmonary artery thromboembolism, acne, decreased BMD, weight gain, and acute urinary retention. No reason for treatment change was recorded in 21.87% of cases.

### 3.4. Symptom Improvement

**Figure 1:**
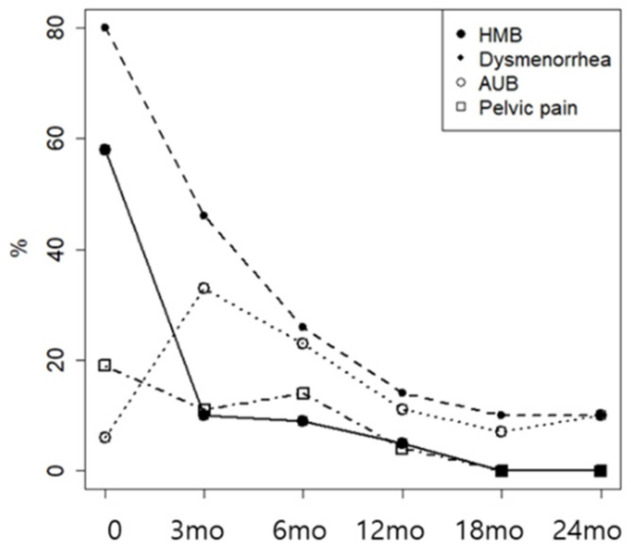
Proportion of patients with each symptom during DNG therapy.

Significant improvements were observed in dysmenorrhea and HMB (Figure 1). Among women with dysmenorrhea, 62 of 78 (79.49%) reported improvement within a median of 5.52 months (mean ± SD, 7.18 ± 5.00 months). Among those with HMB, 53 of 60 (88.33%) improved within a median of 5.36 months (mean ± SD, 5.44 ± 3.34 months). Cox proportional hazards regression analysis was performed to evaluate potential factors associated with the timing of symptom improvement, including age, BMI, baseline uterine volume, initial serum CA-125 level, presence of endometriosis, and primary symptoms. However, no significant associations were identified.

**Figure 2:**
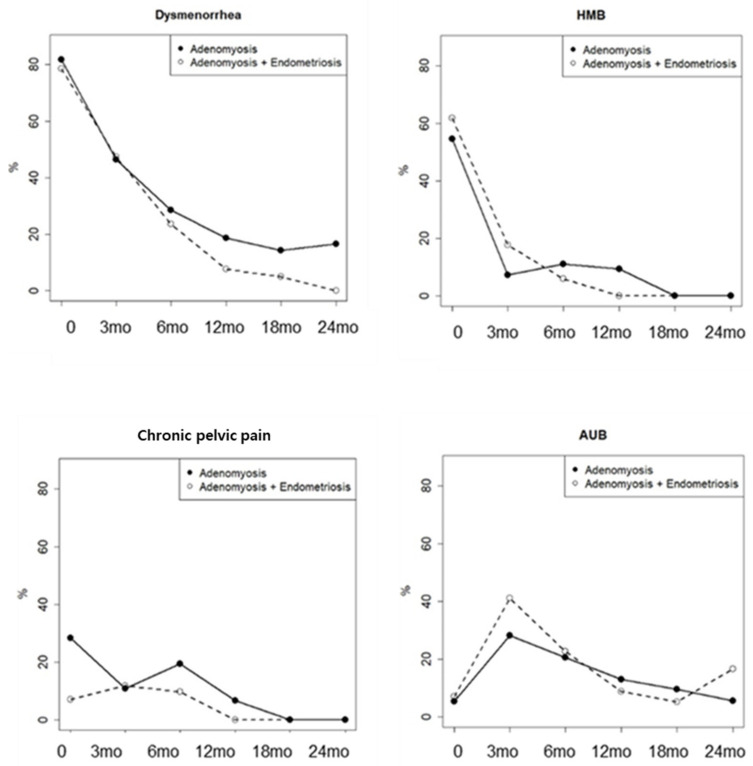
Comparison of proportion of patients with each symptom between patients with adenomyosis with concomitant endometriosis and those without endometriosis symptoms.

Changes in symptom severity were compared between patients with isolated adenomyosis and those with concomitant endometriosis. Although no statistically significant difference was observed between the two groups in terms of dysmenorrhea, a trend toward significance was noted (Figure 2). CA125 levels were also analyzed separately in patients with isolated adenomyosis and in those with concomitant endometriosis, but no statistically significant difference was found between the groups.

### 3.5. Imaging Study and Serum CA-125 Levels

**Figure 3:**
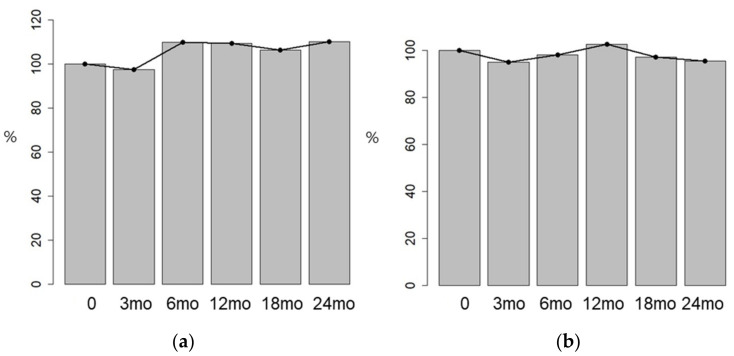
DNG effect on anterior uterine wall thickness (**a**) and posterior uterine wall thickness (**b**)**.**

**Figure 4:**
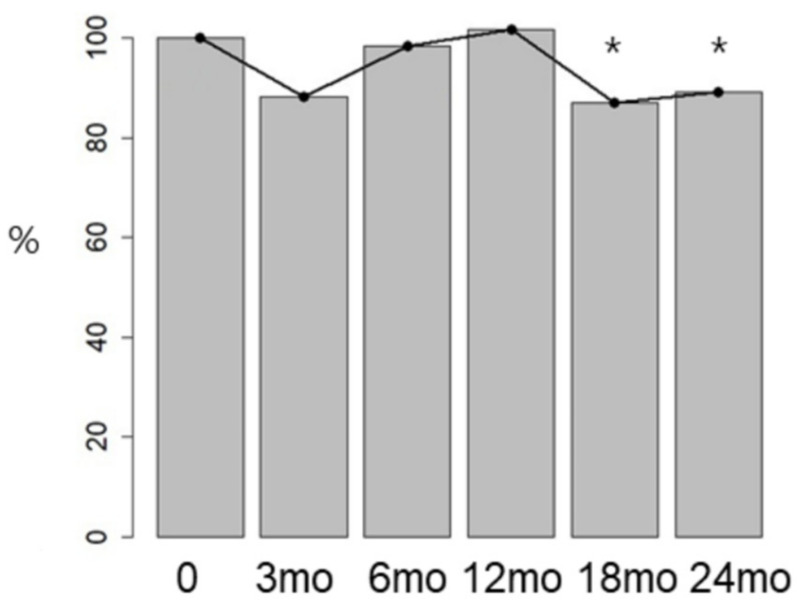
DNG effect on uterine volume (* *p* < 0.05 vs. baseline).

Although anterior and posterior uterine wall thicknesses did not show significant changes over time, uterine volume significantly decreased beginning at 18 months of DNG treatment, demonstrating a 15.10% reduction (31.99 cm^3^) from baseline. The mean uterine volume decreased from 211.75 ± 129.58 cm^3^ at baseline to 179.76 ± 123.85 cm^3^ at 18 months and 183.90 ± 110.11 cm^3^ at 24 months, respectively (Figure 3 and Figure 4).

**Figure 5:**
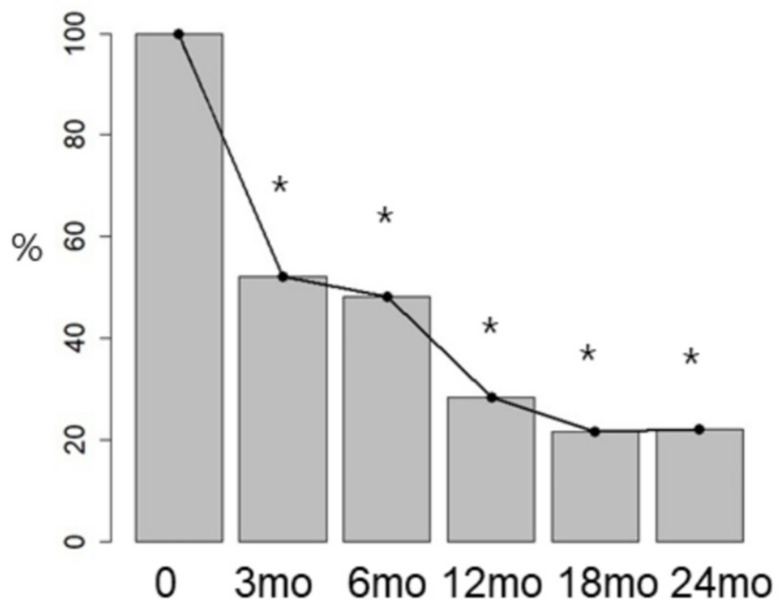
DNG effect on CA 125 level (* *p* < 0.05 vs. baseline).

Serum CA-125 levels also showed a significant decline, with reductions observed as early as 3 months (from 104.86 ± 91.91 U/mL at baseline to 55.31 ± 51.25 U/mL) and normalization by 12 months (30.06 ± 21.47 U/mL). This reduction was sustained through 24 months, decreasing from 104.86 ± 91.91 U/mL at baseline to 19.50 ± 9.36 U/mL at 24 months, respectively (Figure 5).

Spearman correlation analysis showed that symptom improvement during DNG treatment was not significantly associated with imaging improvement, i.e., reduction in uterine volume.

## 4. Discussion

This study demonstrated that DNG improves adenomyosis-related symptoms and leads to a sustained reduction in CA-125 levels beginning at three months of treatment. Long-term treatment led to a significant decrease in uterine volume starting at 18 months, which was maintained up to 24 months. The most common side effect leading to DNG discontinuation was AUB.

Previous studies have similarly reported improvements in pelvic pain, dysmenorrhea, and HMB with DNG in patients with adenomyosis [14,15,17,22,23,24,25,26,27,28,29]. In a meta-analysis including 8 studies of 277 patients treated with the LNG-IUS and 250 patients undergoing DNG therapy, no significant differences were observed between the two groups in terms of improvement in dysmenorrhea, HMB, or changes in uterine size at 3 and 6 months. However, at 12 months, the LNG-IUS group demonstrated a significant reduction in uterine volume, whereas the DNG group showed a significant improvement in dysmenorrhea [28]. Another study also reported that DNG was associated with lower visual analogue scale (VAS) scores compared with LNG-IUS [14]. When compared with COC, both treatment groups demonstrated significant therapeutic efficacy; however, the DNG group showed greater effectiveness, while the incidence of adverse effects—including vaginal spotting, amenorrhea, and hot flushes—was higher in the DNG group [22].

In this study, 55.8% continued DNG, whereas 31.3% changed to alternative therapy. This finding is consistent with a previous study reporting higher discontinuation rate compared with LNG-IUS, although sustained improvement in multiple symptoms over a 3-year period. After the first year, while only 15% of patients using the LNG-IUS switched to alternative treatments, approximately 49% of patients in the DNG group changed therapy due to adverse effects or the need for contraception [26]. Based on these observations, the authors emphasized the need for a variety of progestins with different formulations and routes of administration. In addition to that, the reported presence of progesterone resistance in adenomyosis suggests a potential mechanism underlying the differential therapeutic response observed among patients [19,20]. According to multiple studies, DNG provides equal or greater relief of dysmenorrhea compared to COCs and GnRH agonists, but is less effective in decreasing uterine size and inducing amenorrhea, and is accompanied by a higher rate of side effects [14,15,17,22,23,24,25,26,27].

Findings regarding uterine volume reduction remain inconsistent. Four studies reported no change in uterine volume [23,25,30,31]. Two studies documented decreased uterine volume at 6 and 12 months [22,26]. Uterine volume decreased significantly in the DNG group but not in the COC group (87.65 ± 20.44 vs. 116.22 ± 38.44, *p* = 0.000, respectively) [22]. Significant uterine volume reduction with mean differences of 20.6 ± 30.3% at 24 weeks and 26.0 ± 27.9% at 52 weeks was reported; however, the authors concluded this was statistically but not clinically significant [32]. In a randomized controlled clinical trial comparing LNG-IUS and DNG, DNG demonstrated superior pain relief at 3 months of treatment and noted that although uterine volume decreased at 12 and 24 months, the change was temporary with no significant difference over the entire 72-month period [33]. Conversely, when used for 120 weeks to treat endometriosis, uterine volume decreased after 53 weeks [34]. Most studies have focused on the reduction in uterine volume; however, one study also investigated uterine tissue elasticity and demonstrated significant reductions not only in uterine volume but also in sonographic stiffness [29]. DNG may reduce uterine volume through suppression of estrogen-dependent proliferation, induction of apoptosis in adenomyotic lesions, anti-inflammatory effects, and inhibition of angiogenesis, resulting in adenomyotic tissue regression and myometrial hypertrophy reduction [35,36]. Although uterine volume decreased at 18 months in our findings, the slight increase observed at 24 months is in accordance with prior studies reporting that the volume-reducing effect of DNG may be transient [34]. Longer-term follow-up studies extending beyond this period are warranted to determine whether this reduction is sustained over time or represents a transient effect that plateaus or reverses with prolonged therapy or after treatment discontinuation. In particular, longitudinal assessments are needed to clarify the temporal pattern of uterine volume change and its relationship with symptom recurrence or progression.

Beyond statistical significance, the clinical relevance of uterine volume reduction merits further investigation. A decrease in uterine size may translate into meaningful improvements in compression-related symptoms, such as pelvic heaviness, urinary frequency or urgency, and bowel dysfunction, which are common but often under-evaluated manifestations of adenomyosis. Future studies should therefore incorporate symptom-specific outcome measures to assess whether reductions in uterine volume correlate with improvements in these pressure-related symptoms.

Moreover, uterine volume reduction may have important surgical implications. A smaller uterine size could potentially facilitate surgical procedures by improving operative visibility, reducing technical complexity, and lowering the risk of intraoperative complications, particularly in patients undergoing minimally invasive or uterus-sparing surgery. Evaluating whether preoperative medical therapy–induced uterine volume reduction leads to improved surgical feasibility or outcomes would provide clinically actionable evidence and further support the role of long-term medical management in the treatment strategy for adenomyosis.

Decreased uterine volume has been reported to correlate with CA-125 levels [30,37]. A comparative study of patients with severe adenomyosis demonstrated that larger uterine volumes were associated with higher CA-125 levels, indicating a positive relationship between disease severity (uterine enlargement) and CA-125 [37]. This was compatible with our results showing concurrent decreases in uterine volume and CA-125. The significant CA-125 level reduction following treatment may reflect suppression of adenomyotic lesion activity and inflammation, supporting therapeutic efficacy [30]. Although CA-125 is not routinely used to monitor symptoms in adenomyosis, elevated levels have been associated with disease burden and uterine volume. Therefore, changes in CA-125 may reflect alterations in inflammatory activity rather than direct symptom improvement.

The strengths of this study include, to our knowledge, being the first conducted in a Korean population with a relatively long follow-up period of 24 months. Although no significant correlations were identified, we explored potential factors influencing the timing of symptom improvement, including age, BMI, baseline uterine volume, initial CA-125 level, presence of endometriosis, and primary presenting symptoms. To our knowledge, this is also the first study to longitudinally evaluate changes in anterior and posterior uterine wall thickness, an indirect sonographic feature of adenomyosis according to the MUSA criteria [38]. Study limitations include its retrospective design, lack of a control group, and modest sample size. Although symptom improvement was confirmed, we could not quantitatively assess the extent using VAS scores or comparable scales. Although HMB symptoms improved, pictorial blood assessment chart, hemoglobin level increases, need for iron supplementation, or transfusion requirements could not be confirmed. A limitation of this study is that we did not perform a separate stratified analysis based on the severity of diffuse and focal adenomyosis, which may have provided more detailed clinical insights. Additionally, this study is limited by the absence of a stratified analysis according to the severity of diffuse and focal adenomyosis, which may have provided more detailed clinical implications.

Although DNG has been incorporated into several guidelines for the medical management of adenomyosis [39,40,41], further clinical evidence is still required. Larger-scale studies and randomized controlled trials are needed to clarify its comparative effectiveness relative to other pharmacologic treatments for adenomyosis. Specifically, future research should evaluate the magnitude and time course of symptom improvement for each clinical manifestation, identify clinical risk factors associated with poor symptomatic response, assess the long-term safety of maintenance therapy, and determine the impact of DNG treatment on subsequent fertility outcomes in women desiring pregnancy.

## 5. Conclusions

DNG effectively improved clinical symptoms and reduced uterine volume and serum CA-125 levels without serious adverse events in patients with symptomatic adenomyosis.

## Figures and Tables

**Table 1:** Baseline characteristics.

Characteristic	Value
Age (years, mean ± SD)	39.84 ± 7.01
Body mass index (kg/m^2^, mean ± SD)	22.63 ± 4.26
Parity	
0	52 (50.98%)
1	17 (16.67%)
2	27 (26.47%)
3	5 (4.90%)
No record	1 (0.98%)
Symptoms	
Dysmenorrhea	78 (76.47%)
Heavy menstrual bleeding	60 (58.82%)
Abnormal uterine bleeding	6 (5.88%)
Chronic Pelvic pain	15 (14.70%)
Concomitant or prior endometriosis	44 (43.14%)

SD—standard deviation.

**Table 2:** Previous hormonal treatment.

Treatment	n (%)
Combined oral contraceptives	14 (13.72%)
Gonadotropin-releasing hormone agonist	13 (12.75%)
Levonorgestrel-releasing intrauterine system	14 (13.72%)
None	70 (68.63%)

## Data Availability

The Excel data used to support the findings of this study were supplied by Sa Ra Lee under license, and requests for access to these data should be made to Sa Ra Lee, leesr@amc.seoul.kr.
